# Estimating the Risk of Chronic Pain: Development and Validation of a Prognostic Model (PICKUP) for Patients with Acute Low Back Pain

**DOI:** 10.1371/journal.pmed.1002019

**Published:** 2016-05-17

**Authors:** Adrian C. Traeger, Nicholas Henschke, Markus Hübscher, Christopher M. Williams, Steven J. Kamper, Christopher G. Maher, G. Lorimer Moseley, James H. McAuley

**Affiliations:** 1 Prince of Wales Clinical School, University of New South Wales, Sydney, New South Wales, Australia; 2 Neuroscience Research Australia, Sydney, New South Wales, Australia; 3 Institute of Public Health, University of Heidelberg, Heidelberg, Germany; 4 Hunter Medical Research Institute and School of Medicine and Public Health, University of Newcastle, Callaghan, New South Wales, Australia; 5 The George Institute for Global Health, University of Sydney, Sydney, New South Wales, Australia; 6 Sansom Institute for Health Research, University of South Australia, Adelaide, Australia; Imperial College London, UNITED KINGDOM

## Abstract

**Background:**

Low back pain (LBP) is a major health problem. Globally it is responsible for the most years lived with disability. The most problematic type of LBP is chronic LBP (pain lasting longer than 3 mo); it has a poor prognosis and is costly, and interventions are only moderately effective. Targeting interventions according to risk profile is a promising approach to prevent the onset of chronic LBP. Developing accurate prognostic models is the first step. No validated prognostic models are available to accurately predict the onset of chronic LBP. The primary aim of this study was to develop and validate a prognostic model to estimate the risk of chronic LBP.

**Methods and Findings:**

We used the PROGRESS framework to specify a priori methods, which we published in a study protocol. Data from 2,758 patients with acute LBP attending primary care in Australia between 5 November 2003 and 15 July 2005 (development sample, *n =* 1,230) and between 10 November 2009 and 5 February 2013 (external validation sample, *n =* 1,528) were used to develop and externally validate the model. The primary outcome was chronic LBP (ongoing pain at 3 mo). In all, 30% of the development sample and 19% of the external validation sample developed chronic LBP. In the external validation sample, the primary model (PICKUP) discriminated between those who did and did not develop chronic LBP with acceptable performance (area under the receiver operating characteristic curve 0.66 [95% CI 0.63 to 0.69]). Although model calibration was also acceptable in the external validation sample (intercept = −0.55, slope = 0.89), some miscalibration was observed for high-risk groups. The decision curve analysis estimated that, if decisions to recommend further intervention were based on risk scores, screening could lead to a net reduction of 40 unnecessary interventions for every 100 patients presenting to primary care compared to a “treat all” approach. Limitations of the method include the model being restricted to using prognostic factors measured in existing studies and using stepwise methods to specify the model. Limitations of the model include modest discrimination performance. The model also requires recalibration for local settings.

**Conclusions:**

Based on its performance in these cohorts, this five-item prognostic model for patients with acute LBP may be a useful tool for estimating risk of chronic LBP. Further validation is required to determine whether screening with this model leads to a net reduction in unnecessary interventions provided to low-risk patients.

## Abstract 2

Adrian Traeger and colleagues report the development and validation of a prognostiv model (PICKUP) for estimating risk of developing chronic low back pain.

## Author Summary

**Why Was This Study Done?:**

- A minority of patients who experience an episode of low back pain develop persistent (chronic) pain.
- Offering tests and treatments to all these patients exposes high numbers of low-risk patients to unnecessary intervention, which is very costly and potentially harmful.
- A tool to help healthcare practitioners accurately predict whether a patient with a recent episode of low back pain will develop persistent pain stands to greatly reduce the burden of low back pain on the health system and on patients.

**What Did the Researchers Do and Find?:**

- We developed a five-item screening questionnaire using study data from 1,230 patients with a recent episode of low back pain.
- We tested how well this screening questionnaire could predict the onset of persistent pain in a separate sample of 1,528 patients.
- We found that the screening questionnaire could predict the onset of persistent pain with acceptable levels accuracy (area under the receiver operating characteristic curve = 0.66 [95% CI 0.63 to 0.69]; intercept = 0.55, slope = 0.89).

**What Do These Findings Mean?:**

- This brief, easy-to-use screening questionnaire could help healthcare practitioners and researchers make an early estimate of a patient’s risk of persistent low back pain.
- The screening questionnaire predicted outcome more accurately in patients with low risk scores than in those with high risk scores.
- Screening patients with a recent episode of low back pain could reduce the number of unnecessary interventions provided to low-risk patients.

## Introduction

Low back pain (LBP) is a major global health problem that, compared to all other diseases and health conditions, is responsible for the most years lived with disability, an estimated 80 million years lived with disability in 2010 [1]. The costs of care, investigations, and lost productivity associated with LBP are a significant economic burden for industrialized nations [2]. For example, estimates for treatments alone are US$50 billion per annum in the United States [3] and US$4 billion in the United Kingdom [4]. The impact of LBP can be profound; in Australia, LBP is the leading cause of early retirement [5] and of income poverty in older adults [6].

Although most people with a new episode, or acute, LBP recover in a few weeks or months, around one-quarter of patients who present to primary care develop chronic LBP (pain lasting for longer than 3 mo) [7]. Chronic LBP is the most problematic type of LBP; its prognosis is poor [8], and it accounts for the majority of costs [4,9]. Between 1992 and 2006, the prevalence of chronic LBP in the United States more than doubled [10]. Managing patients with chronic LBP is difficult, and the effects of contemporary interventions are modest at best [11]. An alternative to costly and ineffective management of these patients is secondary prevention, where the goal is to prevent the onset of chronic LBP [12].

An important first step in secondary prevention is to estimate an individual patient’s risk of developing chronic LBP. The Prognosis Research Strategy (PROGRESS) group recently provided a framework for this step, which involves developing and validating prognostic models to determine risk profiles. For these models to be considered clinically useful, they must be easy to use, be able predict outcome with acceptable accuracy, and be validated in external samples. Risk estimates should be well matched to actual outcomes (calibration), higher for individuals who have a poor outcome than for those who do not (discrimination), and informative enough to justify screening compared to “treat all” or “treat none” approaches (net benefit). Estimates from validated models can add valuable information to the clinical decision-making process [13].

Early, accurate prognostic information also provides the opportunity for practitioners to counsel their patients on the necessity of further treatment [14]. Offering tests and treatments to all patients with acute LBP (“treat all” approach) is expensive and risks exposing high numbers of low-risk patients to unnecessary intervention [15]. Overtreatment of conditions such as LBP overburdens healthcare systems and diverts scarce resources away from where they are most needed [16]. Undertreatment of high-risk patients with acute LBP may also be harmful. A “treat none” approach to acute LBP guarantees that a significant proportion will develop chronic LBP and its long-term consequences [7], and wastes an opportunity to intervene early in primary care.

Targeting early intervention according to risk profile has been shown to be effective in breast cancer [17] and cardiovascular disease [18] and has been identified as a research priority for managing LBP [19]. There is preliminary evidence that a stratified approach improves disability in samples with predominantly chronic LBP [20], but it remains unknown whether such a prognostic approach can prevent the onset of chronic LBP. The absence of a valid prognostic model to inform risk-stratified management of acute LBP is therefore an important area of uncertainty [21]. Not having a validated prognostic model for acute LBP is also problematic for secondary prevention trials that are designed to target pain [22,23]; treat all approaches are unlikely to be efficient if the majority of included participants are at low risk of chronic LBP [24,25].

None of the commonly used screening tools in LBP are suited to this purpose. Tools such as the Start Back Tool (SBT) and the Orebro Musculoskeletal Pain Questionnaire (OMPQ) were either developed in samples that included patients with chronic LBP [26] or used to predict disability [26,27] or return to work [28] outcomes. When these tools were subsequently tested in acute LBP samples, they predicted chronic LBP with modest accuracy at best [27,29–31].

The primary aim of this study was to develop and validate a prognostic model to identify risk of chronic LBP in patients with acute LBP. Specifically, we aimed to develop a model that can provide an estimate for an individual patient’s risk of chronic LBP with acceptable levels of accuracy (calibration, discrimination, and net benefit). A secondary aim was to determine whether prognostic models varied by how chronic LBP was defined. Specifically, we aimed to develop two additional prognostic models using outcomes of high pain and chronic disability.

## Methods

The protocol for this study has been published [32].

### Patients

We used patient data from a prospective cohort study to develop the model (development sample) and patient data from a randomized trial to externally validate the model (external validation sample). Full details of these two studies have been published [23,33], and their key differences are summarized in Table 1. Both studies were conducted in Sydney, Australia. In short, the cohort study recruited consecutive patients with acute LBP presenting to their primary care provider (general practitioner, physiotherapist, chiropractor) between 5 November 2003 and 15 July 2005. The randomized trial recruited consecutive patients with acute LBP presenting to their primary care practitioner between 10 November 2009 and 5 February 2013 to test the effect of paracetamol on recovery. There was no difference in treatment effects between groups. Both studies followed a published protocol [34,35], and the trial was prospectively registered.

**Table 1:** Key differences in the development and external validation studies.

Characteristic	Development Sample	External Validation Sample
**Design**	Cohort study	Randomized trial
**Recruitment period**	2003–2005	2009–2013
**Number of participants by recruitment setting**		
Physiotherapy	77	4
General practice	73	181
Pharmacy	0	50
Chiropractic	20	0
**Location**	Sydney metropolitan area	Greater Sydney area
**Inclusion/exclusion criteria**		
Radiculopathy	Excluded	Included
Moderate intensity pain	Not considered	Included
Use of regular analgesics	Included	Excluded
**Treatment**	Advice plus usual care	Advice plus paracetamol or placebo

### Predictors and Model Outcomes

Baseline data were available on 20 predictors in six broad groups of putative prognostic factors that have been identified in previous studies [36–38]: sociodemographic factors, general health, work factors, current LBP characteristics, past LBP history, and psychological factors. Primary care clinicians collected these data at the first consultation. A full list of individual candidate predictors is provided in Table 2.

**Table 2:** Candidate predictors.

Prognostic Factor Group	Characteristic	Question (Measure)
**Sociodemographic factors**	Age	What year were you born? (year)
	Gender	What is your gender? (male/female)
	Education level	What is the level of the highest qualification you have completed? (school certificate/higher school certificate/trade certificate/diploma/advanced diploma/bachelor degree/postgraduate degree/other)
**Current LBP characteristics**	Duration of LBP episode	How long ago did the present episode of low back pain begin? (<2 wk/2–3 wk/3–4 wk/4–6 wk)
	Sudden onset	Was the onset of low back pain sudden? (yes/no)
	Leg pain	Do you have leg pain? (yes/no)
	Pain intensity	How much low back pain have you had during the past week? (none/very mild/mild/moderate/severe/very severe)
	Interference of symptoms	During the past week, how much did low back pain interfere with your normal work (including both work outside the home and housework)? (not at all/a little bit/moderately/quite a bit/extremely)
	Medication	Are you currently taking medication for your low back pain? (yes/no)
**Past LBP history**	Previous episodes	Have you had a previous episode of low back pain? (yes/no)
	Surgery	Have you previously had surgery for low back pain? (yes/no)
**Psychological factors**	Control of pain	Based on all the things you do to cope, or deal with your pain, on an average day, how much are you able to decrease it? (0–10 scale)
	Anxiety	How tense or anxious have you felt in the past week? (0–10 scale)
	Depression	How much have you been bothered by feeling depressed in the past week? (0–10 scale)
	Perceived risk	In your view, how large is the risk that your current pain may become persistent? (0–10 scale)
**General health**	Smoking	Do you currently smoke? (yes/no)
	Exercise	At the commencement of this back pain episode were you exercising for at least 30 minutes three times per week or more (exercise includes walking briskly, cycling, digging, scrubbing floor on hands and knees, etc.)? (yes/no)
	Perceived general health	In general how would you say that your health is? (excellent/very good/good/fair/poor)
**Work factors**	Sick leave	Have you previously taken sick leave due to low back pain? (yes/no)
	Disability compensation	Is your back pain compensable, e.g., through worker’s compensation or third party insurance? (yes/no)

To develop the primary model, PICKUP (Predicting the Inception of Chronic Pain), we defined the main outcome as whether or not patients had chronic LBP, that is, ongoing LBP 3 mo after the initial consultation. In the development study, pain intensity was measured with a six-point Likert scale [39]. We classified patients as having “chronic LBP” if they reported greater than “mild” (2 on the Likert scale) pain intensity at 3-mo follow-up and had no periods of recovery [40].

To develop two secondary prognostic models (Models 2a and 2b), we used additional criteria to define chronic LBP. These secondary models allowed comparison of model performance to published models and to our primary prognostic model. Patients were classified as having “chronic LBP high pain” if they reported greater than “moderate” (3 on the Likert scale) pain intensity [39] at 3-mo follow-up (Model 2a). Patients were classified as having “chronic LBP disability” if they reported a score of 2 or more on a five-point Likert scale for disability [39] at 3-mo follow-up (Model 2b). Thresholds to define outcomes for all three models were determined a priori [32].

In the external validation sample, pain and disability scores were converted from an 11-point scale used to measure pain intensity and a 24-item scale used to measure disability to the six-point and five-point scales, respectively, used in the development sample. Both of the original studies assessed 3-mo outcomes over the phone, an approach that yields comparable results to in person assessment on pain-related outcomes [41].

### Statistical Analysis

The statistical analysis plan for this study was informed by recommendations from the PROGRESS group [13]. All preplanned analyses are outlined in our protocol published a priori [32].

#### Missing data

We planned a complete case analysis if less than 5% of predictor values were missing. If more than 5% of predictor values were missing, we planned to impute the missing values. Because PROGRESS does not recommend a complete case analysis, we performed a post hoc sensitivity analysis using the Expectation Maximization algorithm in SPSS to impute missing values and to test the robustness of our approach. We did not impute missing outcome values [42].

#### Model specification

To identify predictors in the development sample, we performed a forward stepwise logistic regression analysis. We set the significance level for variable selection at *p <* 0.10. To specify the model, age, sex, and duration of the pain episode were forced into the first block, and the remaining candidate predictors (Table 2) were selected using an automated stepwise procedure in the second block. Only those predictors identified in the second block using the stepwise procedure were included in the final models for external validation. We examined the linearity of continuous predictor variables using scatter plots and Box–Tidwell transformations [43].

#### Performance measures

We assessed the predictive performance of the regression model by examining measures of discrimination, calibration, and overall performance. Discrimination refers to how likely the model is to allocate higher predicted risks to patients who develop chronic LBP during the study period and lower predicted risks to those who do not. We assessed discrimination by calculating the area under the receiver operating characteristic curve (AUC) [44]. With this statistic, a value of 0.5 indicates that the model discriminates no better than chance and a value of 1 indicates that the model discriminates perfectly [45]. We further assessed discrimination by calculating the discrimination slope (the absolute difference in mean predicted risk in those who developed chronic LBP and those who did not) [46] and risk-stratified likelihood ratios.

Calibration refers to the agreement of predicted risks and actual outcomes. In both samples, we constructed calibration graphs that plotted predicted risks produced from the prognostic model versus observed proportions of chronic LBP in ten groups separated by decile of risk. We fitted a smoothed line to the calibration graph to calculate the calibration slope and intercept; values around 1 for the slope and 0 for the intercept represent correct calibration [46].

Overall performance and model fit indices combine aspects of discrimination and calibration. We tested overall performance using the Nagelkerke *R* ^2^ statistic and the Brier score. Nagelkerke *R* ^2^ measures the additional variation in chronic LBP that is explained by the model compared to an intercept-only logistic model. A large difference in Nagelkerke *R* ^2^ between the development and external validation samples indicates overfitting and poor generalizability [46]. The Brier score quantifies the average prediction error and ranges from 0 to 0.25; values close to 0 represent informative models, while values close to 0.25 represent non-informative models [47].

#### Internal validation (development sample)

Prediction models tend to perform optimistically (i.e., overestimate performance) in the sample in which they are developed. To provide a robust estimate of model performance in the development sample, we bootstrapped all of the performance estimates according to Harrell et al. [48]. Bootstrapping is the most efficient method of internally validating performance estimates in a development sample [49]. In brief, this procedure creates bootstrap samples by drawing random samples with replacement from the development sample (200 replications) and then tests model performance in the newly created sample. This allows performance estimates in the development sample to be adjusted for optimism. Although we planned to use SPSS to perform the bootstrap procedure [32], we found it to be easier to perform using R software with the syntax provided by Steyerberg [42].

#### External validation (external validation sample)

To externally validate the model, we tested model predictions in the external validation sample and calculated the performance statistics described above. To update the model, we examined whether including a recently identified prognostic factor—sleep quality [50,51]—added significantly (*p <* 0.10) to the model. All models were recalibrated according to the method of Steyerberg [42], which involved updating the logistic equation using the calibration slope and intercept obtained in the external validation procedure.

#### Clinical usefulness

We assessed the potential clinical utility of the model by selecting cutoffs based on quartiles of predicted risk in the development sample. Predicted risk, or predicted probability, is calculated using the regression equation and produces a number between 0 and 1. For example, a predicted probability of 0.2 signifies a 20% (absolute) predicted risk of developing chronic LBP. Those in the highest quartile of predicted risk were classified as high risk, those in the middle two quartiles as medium risk, and those in the lowest quartile as low risk. Using these cutoffs, we calculated posterior probabilities and likelihood ratios with 95% confidence intervals.

To further explore clinical utility, we performed a decision curve analysis. This analysis allowed us to assess whether using a prognostic model to screen patients could be a superior decision-making approach to simply intervening with all patients (treat all approach) or intervening with none (treat none approach). The decision curve analysis calculates the net benefit of a particular decision-making approach across a range of risk thresholds where patients and their physicians might opt for further intervention. For example, a physician may decide to recommend further intervention (e.g., a course of physiotherapy) for cases with a greater than 30% risk of chronic LBP. A decision curve analysis estimates whether this approach would provide a net increase in the proportion of patients treated appropriately (i.e., patients with a poor prognosis are recommended further intervention, those with a good prognosis are not). Specifically, the net benefit is the difference in proportions of true positives and false positives when false positives are weighted by the odds of the selected cutoff (net benefit = TP–*w*FP/*N*, where TP is the number of true positive decisions, *w*FP is the number of false positive decisions × the odds of a given cutoff, and *N* is the total number of patients).

We also calculated the net number of unnecessary interventions avoided using the equation suggested by Vickers and Elkin [52]:

(net benefit of the model − net benefit of treat all)/(threshold probability/[1 − threshold probability])×100

This equation estimates the net number of unnecessary interventions that would be avoided if clinicians were to base their decision to recommend further intervention on predicted risks. For example, compared to treat all, what is the net number of unnecessary interventions that would be avoided if only those with 30% or higher risk of chronic LBP were recommended further intervention? We calculated, across a range of potential cutoff scores, the net reduction in the number of patients with good outcomes who would receive unnecessary interventions using a treat all strategy.

Statistical analyses were carried out in SPSS Statistics for Windows version 22.0 (IBM Corp) and R version 3.1.2 [53].

Both of the original studies were approved by the Human Research Ethics Committee of the University of Sydney (ref 11-2002/3/3144 and ref 11638). All participants provided written informed consent. Because we analyzed an existing non-identifiable dataset, the Human Research Ethics Committee did not require a separate ethics application for the current study. A non-identifiable dataset is provided in S1 and S2 Data.

## Results

**Fig 1:**
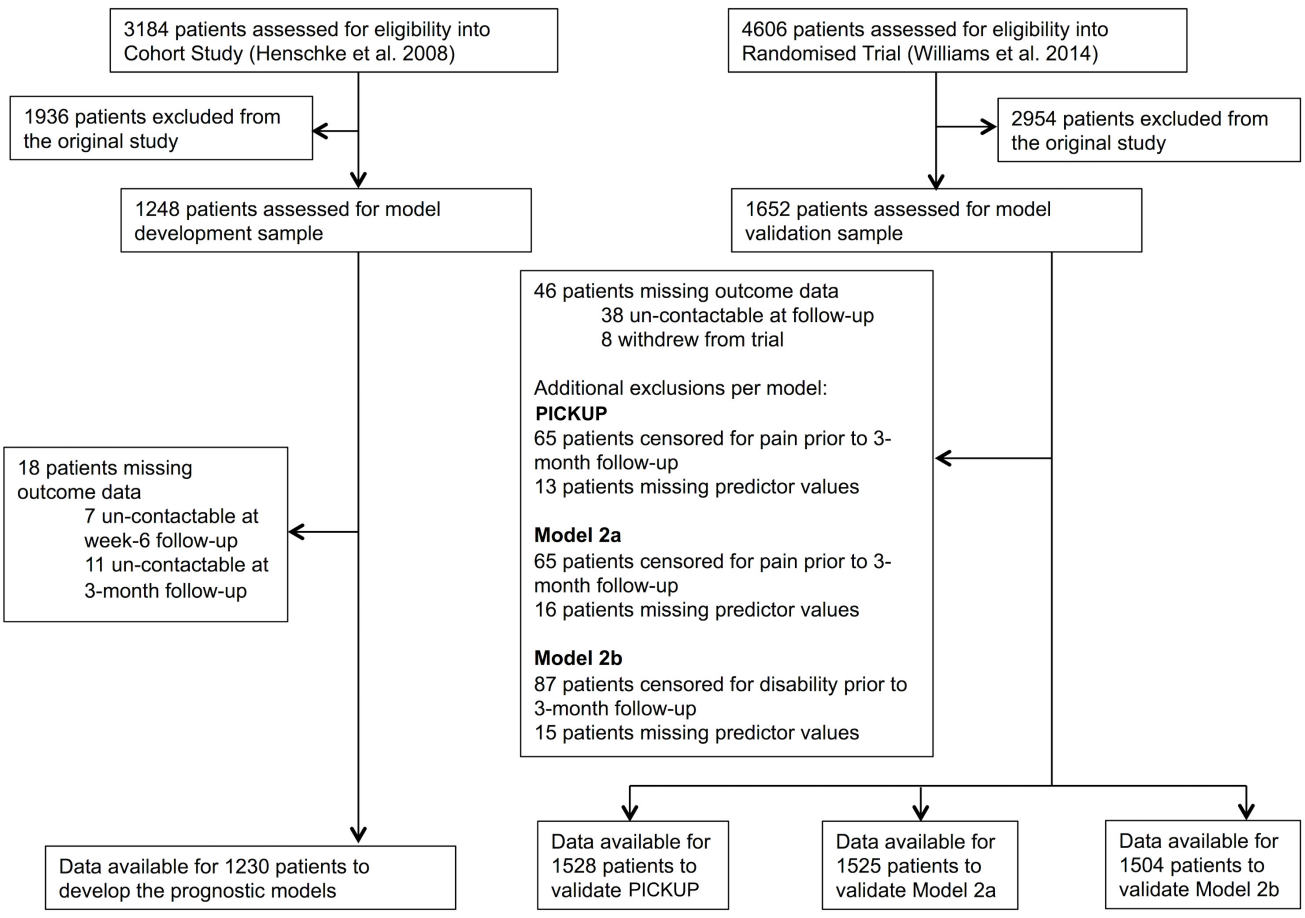
Patient flow chart. The current study used non-identifiable data originally published in Henschke et al. [33] (development sample) and Williams et al. [23] (external validation sample).

Flow of patients in the development and external validation samples is shown in Fig 1. Eighteen patients (1.4%) in the cohort study (development sample) and 46 patients (2.7%) in the randomized trial (external validation sample) were un-contactable at 3-mo follow-up. Some patients were excluded from the external validation sample because they were not assessed for pain intensity (65 patients; 3.9%) or disability (87 patients; 5.2%) at 3-mo follow-up.

There were five missing predictor values in the development sample and 44 missing predictor values in the external validation sample. We found evidence against the hypothesis that predictor values were not missing completely at random (Little’s test, *p* > 0.05), and, because the number of missing values was small (<1%), we removed these cases from the primary analysis as per our protocol [32]. Imputing missing predictor values in the sensitivity analysis did not affect the results (S1 and S2 Tables).

Data were therefore available from 1,230 cases to develop the prognostic models. To externally validate the models, data were available from 1,528 complete cases to test PICKUP, 1,525 complete cases to test Model 2a, and 1,504 complete cases to test Model 2b.

Table 3 shows the characteristics of patients in the development and external validation samples. Patients were similar at baseline except for the proportion receiving disability compensation, which was higher in the development sample (18%) than in the external validation sample (7%).

**Table 3:** Patient characteristics in the development and external validation samples.

Factor Group/Outcome	Characteristic	Development Sample (*n =* 1,230)	External Validation Sample (*n =* 1,528)
**Sociodemographic factors**	**Age, years**	44 (14.8)	45 (15.8)
	**Female gender**	572 (46%)	706 (46%)
	**Born in Australia**	856 (70%)	—
	**Aboriginal/Torres Strait Islander**	8 (<1%)	—
	**Bachelor degree or higher education**	322 (26%)	—
**Current LBP characteristics**	**Duration of LBP episode**		
	Less than 2 wk	989 (80%)	1,183 (78%)
	2 to 3 wk	171 (14%)	149 (10%)
	3 to 4 wk	70 (6%)	77 (5%)
	4 to 6 wk	0 (0%)	116 (8%)
	**Sudden onset**	971 (79%)	—
	**Leg pain**	292 (24%)	294 (19%)
	**Pain intensity**		
	None	3 (<1%)	0 (0%)
	Very mild	28 (2.3%)	290 (19%)
	Mild	112 (9%)	242 (16%)
	Moderate	458 (37%)	565 (37%)
	Severe	529 (43%)	346 (23%)
	Very severe	100 (8%)	70 (5%)
	**Interference of symptoms**		
	Not at all	74 (6%)	0 (0%)
	A little bit	191 (16%)	229 (15%)
	Moderately	290 (24%)	320 (21%)
	Quite a bit	460 (37%)	488 (32%)
	Extremely	215 (18%)	412 (27%)
	**Currently taking pain medication**	498 (41%)	590 (39%)
**Past LBP history**	**Previous episodes**	911 (74%)	1,095 (72%)
	**Surgery**	32 (2%)	—
**Psychological factors**	**Control of pain (0–10 scale)**	4.8 (2.5)	—
	**Anxiety (0–10 scale)**	5.5 (2.6)	4.8 (2.2)
	**Depression (0–10 scale)**	3.3 (3.1)	3.1 (2.9)
	**Perceived risk (0–10 scale)**	4.5 (2.9)	4.5 (2.8)
	**Satisfaction with symptoms**		
	Very dissatisfied	920 (74.8%)	—
	Somewhat dissatisfied	241 (20%)	—
	Neither satisfied nor dissatisfied	34 (2%)	—
	Somewhat satisfied	13 (<1%)	—
	Very satisfied	22 (2%)	—
**General health**	**Smoking**	231 (19%)	—
	**Exercise 30 min 3×/week**	702 (57%)	—
	**Perceived general health**		
	Excellent	210 (17%)	222 (15%)
	Very good	506 (41%)	553 (36%)
	Good	417 (34%)	565 (37%)
	Fair	90 (7%)	156 (10%)
	Poor	7 (<1%)	25 (2%)
**Work factors**	**Sick leave**	462 (38%)	—
	**Disability compensation**	225 (18%)	107 (7%)
**Outcomes**	**Chronic LBP**	371 (30%)	291 (19%)
	**Chronic LBP high pain**	217 (18%)	162 (10%)
	**Chronic LBP disability**	380 (31%)	217 (14%)

All values are given as number (percentage of total) or mean (standard deviation). Cells marked with a dash (—) indicate that the variable was not measured.

### Model Development and Internal Validation

At 3 mo, 30% of the patients in the development sample were classified as having chronic LBP. Table 4 shows predictors and regression coefficients for the primary model (PICKUP) and the two secondary models that were fitted in this sample. PICKUP contained five predictors. We did not detect significant non-linearity in any continuous predictor variables. Estimates for the predictive performance of each prognostic model in the development sample can be found in S2 Table. Recruitment setting (general practice, physiotherapy, chiropractic) did not affect performance estimates (S3 Table).

**Table 4:** Predictors and regression coefficients for the three prognostic models.

Predictor	PICKUP		Model 2a		Model 2b	
	Regression Coefficient	Odds Ratio (95% CI)	Regression Coefficient	Odds Ratio (95% CI)	Regression Coefficient	Odds Ratio (95% CI)
Disability compensation(yes/no)	0.50	1.65 (1.20 to 2.25)	0.42	1.52 (1.06 to 2.18)	0.43	1.53 (1.12 to 2.09)
Leg pain (yes/no)	0.44	1.56 (1.17 to 2.08)	0.53	1.71 (1.23 to 2.38)	0.46	1.58 (1.18 to 2.10)
Pain intensity (1–6 scale)	0.21	1.23 (1.06 to 1.44)	0.28	1.32 (1.09 to 1.60)	0.25	1.29 (1.10 to 1.50)
Depression (0–10 scale)	0.06	1.06 (1.02 to 1.11)	NS	NS	0.07	1.07 (1.03 to 1.12)
Perceived risk (0–10 scale)	0.13	1.14 (1.09 to 1.20)	0.14	1.15 (1.09 to 1.22)	0.11	1.12 (1.07 to 1.17)
Medication use (yes/no)	NS	NS	0.40	1.49 (1.08 to 2.05)	NS	NS
General health (1–5 scale)	NS	NS	NS	NS	0.25	1.28 (1.10 to 1.48)
Constant	−2.82		−3.92		−3.49	

Values are adjusted for age, gender, and duration of LBP episode.

NS, non-significant predictor.

### External Validation

Table 5 summarizes the predictive performance of the prognostic models in the external sample. At 3 mo, 19% of the patients in the external validation sample were classified as having chronic LBP. The Nagelkerke *R* ^2^ value was 7.7%, compared to 10.9% in the development sample, and the Brier score was 0.15, indicating a similar overall model fit. S2 Table shows the full results of performance testing for each prognostic model in the development and external validation samples. Discrimination performance for PICKUP fell within our prespecified acceptable range: the AUC was 0.66 (95% CI 0.63 to 0.69), the likelihood ratio in the high-risk group was 2.99 (95% CI 2.81 to 3.18), and the 95% confidence intervals did not overlap with between risk groups (S4 Table).

**Table 5:** Summary performance measures in the external validation sample.

Aspect	Measure	PICKUP (*n =* 1,528)	Model 2a (*n =* 1,525)	Model 2b (*n =* 1,504)
Overall Performance	*R* ^2^ (Nagelkerke)	7.7	4.8	10.1
Discrimination	AUC	0.66 (0.63 to 0.69)	0.64 (0.60 to 0.68)	0.69 (0.64 to 0.72)
Calibration	Calibration intercept	−0.55	−0.81	−0.86
	Calibration slope	0.89	0.74	0.99
Decision curve analysis	Net benefit at incidence rate cutoff^a^	0.04	0.06	0.04
	Net number of unnecessary interventions avoided at 30% risk cutoff^b^	46	54	52

^a^The proportion of patients with poor outcomes who would correctly be recommended further intervention at the same rate of not recommending intervention for patients with good outcomes, when the threshold probability is set at the incidence rate in the external validation sample (i.e., 19% for PICKUP, 10% for Model 2a, 14% for Model 2b).

^b^Net number of unnecessary interventions avoided per 100 acute LBP patients without missing any patients who developed chronic LBP, if only patients with predicted risks higher than the cutoff are recommended further intervention.

**Fig 2:**
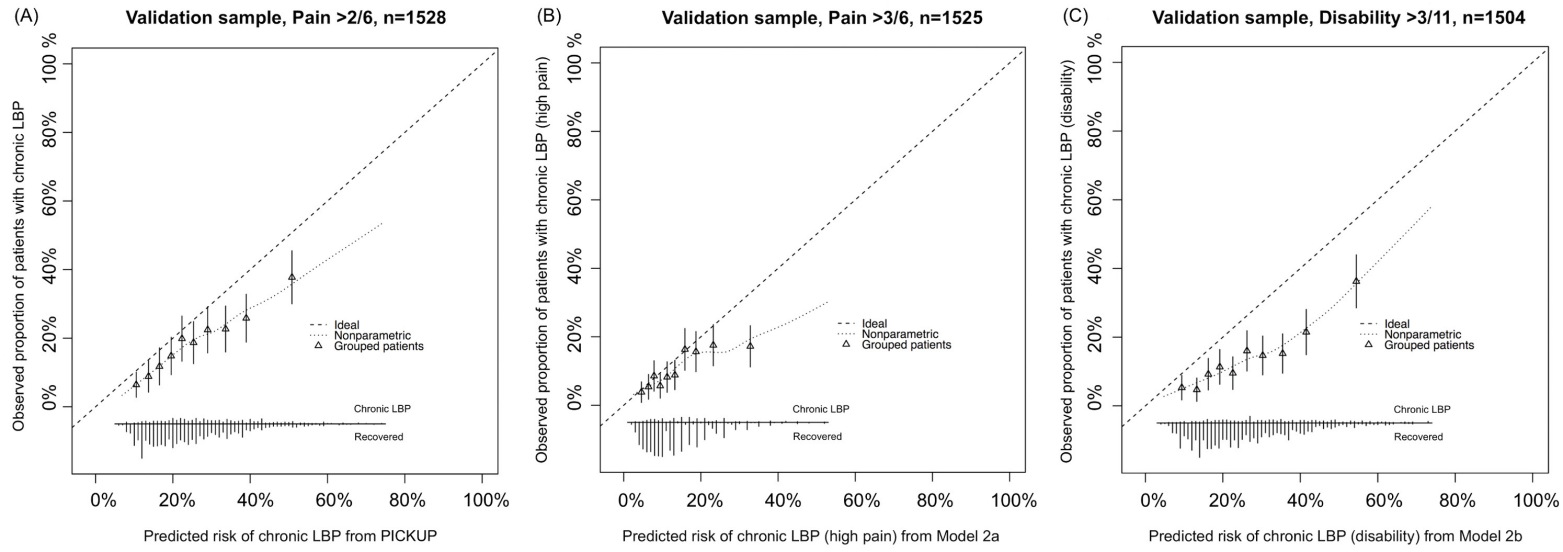
Calibration plots showing external validity of the three prognostic models. (A) PICKUP predicting chronic LBP. (B) Model 2a predicting chronic LBP with high pain. (C) Model 2b predicting chronic LBP with disability. The distribution of predicted risks is shown at the bottom of each plot, by 3-mo outcome. The triangles indicate observed frequencies by decile of predicted risk.

All models showed some miscalibration in the external validation sample (Fig 2). PICKUP demonstrated the best calibration and fell within our prespecified acceptable range in the lower seven of the ten risk groups, that is, predictions were within 5% of actual proportions of chronic LBP. In all three models, calibration was better for the low-risk patients than it was for the high-risk patients. After recalibration, slope and intercept estimates for each model were close to 1 and 0, respectively, which indicates near perfect calibration (S1–S3 Figs). Updating PICKUP with an additional prognostic factor (sleep quality) did not add significantly to the model (*p* > 0.10).

### Clinical Usefulness

**Fig 3:**
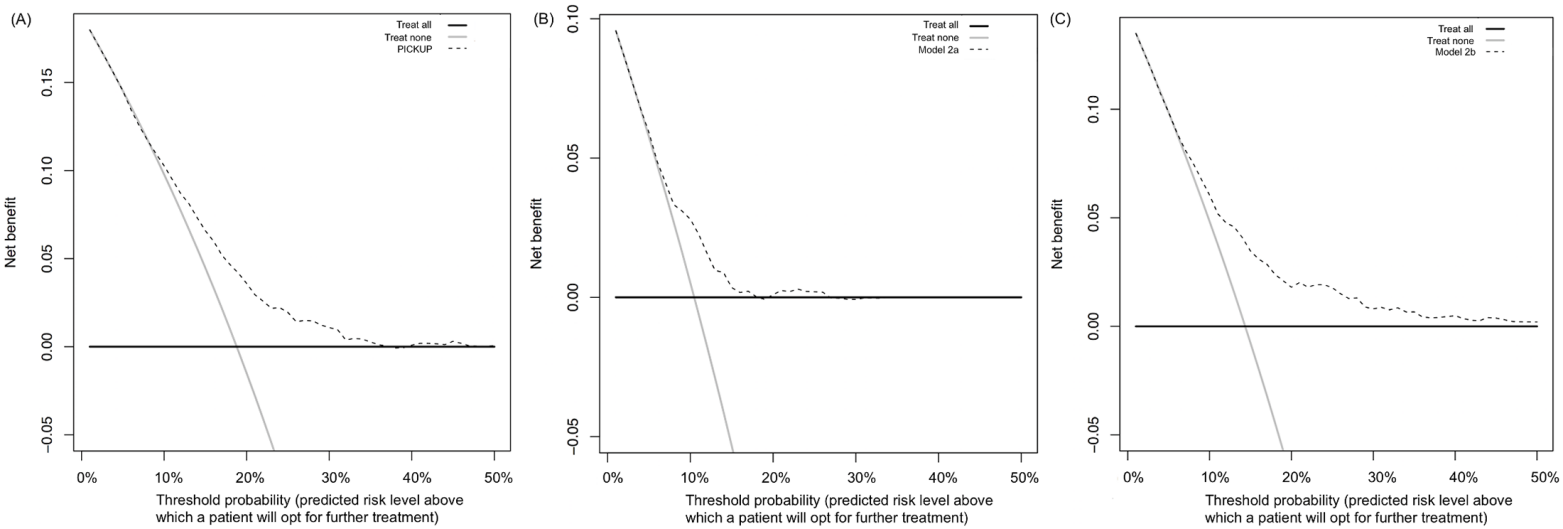
Decision curve analysis for the three prognostic models in the external validation sample. Net benefit of using PICKUP (A), Model 2a (B), or Model 2b (C) as a decision strategy. The net benefit (*y*-axis) is the net proportion of patients with poor outcomes who, based on the decision strategy, would correctly be recommended further intervention at the same rate that patients with good outcomes would not be recommended further intervention. The threshold probability (*x*-axis) indicates the range of predicted risk levels above which patients and their physicians might opt for further intervention. A threshold probability of 10% implies that a patient or physician would opt for further intervention if the predicted risk of chronic LBP was higher than 10%. The decision curve analysis estimates the net benefit of screening at all possible thresholds. On the plots, the line that is the highest over the widest range of thresholds indicates the strategy with the highest net benefit. For PICKUP (A), there is little difference in net benefit between the treat all strategy (grey line) and screening (dashed line) at cutoffs between 0% and 10%. At cutoffs between 12% and 35% predicted risk, screening with PICKUP would produce the highest net benefit. Treating none always yields a net benefit of 0 (black line). The highest net benefit usually occurs at the incidence of the outcome, in this case at a threshold probability of 19%.

Fig 3 shows the results of the decision curve analysis. Treat all strategies assume that if all patients are treated, none will develop an unfavorable outcome. This may or may not be a reasonable assumption in LBP. Although there are effective treatments for acute LBP [54], evidence-based interventions to prevent the onset of chronic LBP are not yet available. The assumed outcome from treating all patients with acute LBP is that all high-risk patients are offered further intervention that could reduce their risk of chronic LBP. The assumed outcome from treating no patients with acute LBP is that all high-risk patients will develop an unfavorable outcome. In our external validation cohort, for example, if no high-risk patients were offered further intervention, one in five would develop chronic LBP.

Treat all strategies demonstrated the highest net benefit at threshold probabilities between 0% and 10%. At thresholds above the population risk (incidence rates were 19% for chronic LBP, 10% for chronic LBP with high pain, and 14% for chronic LBP with disability), the net benefit of treating all became negative (Fig 3). The net benefit of treating none was always assumed to be zero.

All prognostic models showed equal or higher net benefit than the treat all and treat none strategies. Using PICKUP and a cutoff set at 19% (i.e., only patients with a predicted risk higher than the population risk of 19% are recommended further intervention), the net number of cases of chronic LBP that would be detected through screening, without any increase in the number of patients unnecessarily recommended further intervention, would be four in every 100 patients.

**Fig 4:**
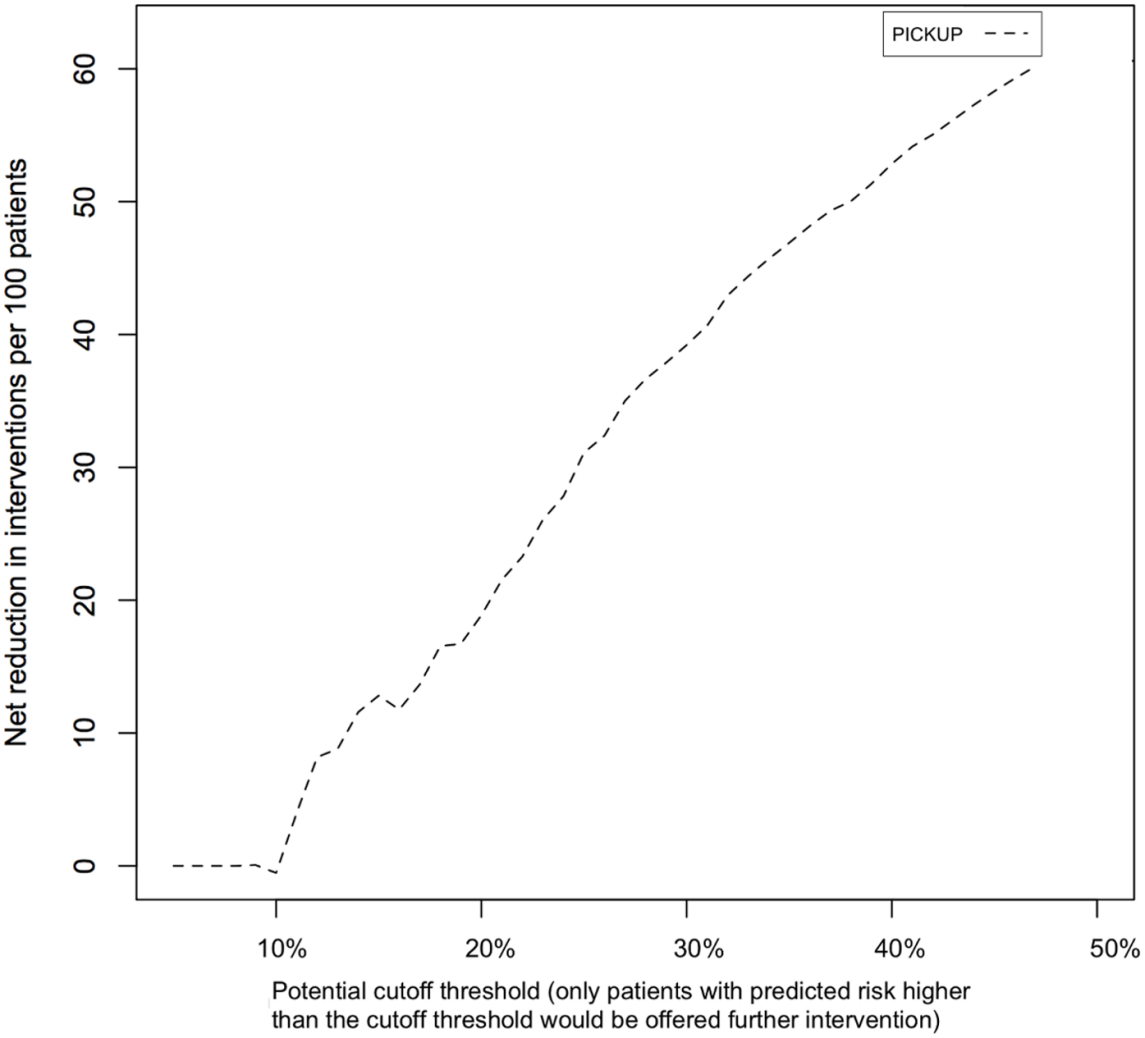
Net number of unnecessary interventions avoided if patients in the external validation sample were screened using PICKUP. The net reduction (*y*-axis) is the number of unnecessary interventions avoided without missing any patients who develop chronic LBP. The cutoff threshold (*x*-axis) is the range of potential predicted risk cutoffs where a patient or physician would decide to pursue further intervention.

Fig 4 shows the estimated net number of unnecessary interventions avoided through screening. Using PICKUP and a cutoff set at 30% (i.e., only patients with a predicted risk of 30% or higher are recommended further intervention) would lead to a net reduction of around 40 unnecessary interventions per 100 patients.

### Calculation of an Individual Predicted Risk

An individual score (ScoreCLBP) can be derived using the recalibrated logistic regression equation from PICKUP:

ScoreCLBP=−0.55 + 0.89*(−2.82 + [0.21*Pain + 0.44*Leg + 0.50*Comp + 0.06*Depress + 0.13*Risk])

where Pain = “How much low back pain have you had during the past week?” 1 = none, 2 = very mild, 3 = mild, 4 = moderate, 5 = severe, 6 = very severe; Leg = “Do you have leg pain?” 0 = no, 1 = yes; Comp = “Is your back pain compensable, e.g., through worker’s compensation or third party insurance?” 0 = no, 1 = yes; Depress = “How much have you been bothered by feeling depressed in the past week (0–10 scale)?” 0 = not at all, 10 = extremely; Risk = “In your view, how large is the risk that your current pain may become persistent (0–10 scale)?” 0 = none, 10 = extreme.

The predicted risk of developing chronic LBP (ProbCLBP) can then be calculated using the score and the following equation:

ProbCLBP = exp(ScoreCLBP)/(1 + exp[ScoreCLBP])

## Discussion

We have developed and tested the external validity of a prognostic model to identify the risk of chronic LBP in individuals with acute LBP. Values for discrimination and calibration fell within a prespecified [32] range of what we subjectively determined to be informative. Although the AUC values are modest (between 0.66 and 0.69), they suggest better predictive accuracy for pain outcomes than recently published values based on either clinician judgment alone (between 0.50 and 0.60) [29] or popular tools such as the SBT and OMPQ [29–31]. The results of our decision curve analysis indicate that, compared to treat all and treat none strategies, our model has the potential to substantially reduce harms associated with undertreating high-risk patients and overtreating low-risk patients with acute LBP.

The major strengths of this study are its preplanned methods, the use of large, high-quality datasets, and transparent reporting. To our knowledge, this is the largest “Type 3” study in LBP to have—in line with the PROGRESS initiative [13]—published a statistical analysis plan and reported results using the TRIPOD statement (see S1 TRIPOD Checklist). Type 3 studies build on foundational prognostic factor research (Type 1 and 2 studies) [55] by constructing prognostic models. Constructing accurate prognostic models is an essential step towards improving patient outcomes through stratified care (Type 4 studies) [56]. We used large samples of patients with acute LBP to develop and externally validate the models. The samples had a number of differences (Table 1), not least of which was the overall risk of developing chronic LBP (30% in the development sample versus 19% in the external validation sample). Despite these differences, the models made informative predictions in the external sample, which indicates favorable generalizability and suggests that further testing in additional samples is warranted. We have reported different aspects of model performance that can be interpreted for clinical and research applications.

This study has some limitations. First, we were restricted to the use of predictor variables measured in the original studies. We were therefore not able to directly compare our model or update existing models in this study, as is recommended by the PROGRESS framework (Recommendation 21) [13]. Moreover, we may not have included important prognostic variables in our models because they were not measured in the original studies. We attempted to overcome this limitation by updating the model at the external validation stage. Interestingly, when we updated the model with a recently identified prognostic factor, sleep quality [50,51], there was no improvement in any of our indices of predictive performance. Second, we used an automated stepwise approach to specify the models, principally because it is objective and generally results in smaller, clinically applicable models [57], but stepwise methods have well-known limitations such as unstable variable selection [58] and biased coefficient estimation [57]. It is therefore conceivable that our choice to use stepwise selection may have reduced the predictive performance of the models. Third, the overall model fit statistics indicate that the variance explained by our prediction models is modest. Perhaps some factors that are yet to be tested thoroughly in LBP, for example, structural pathology shown on imaging [59], explain additional variance in chronic LBP. However, tests involving imaging are onerous, costly, and potentially harmful for patients with acute nonspecific LBP [60]. Fourth, by prespecifying in our protocol that we would impute missing predictor values only if they were missing in more than 5% of the sample, we did not strictly adhere to the PROGRESS recommendation to impute values where reasonable (Recommendation 20). The complete case approach that we used in our primary analysis can be inefficient and is known to produce bias in prediction research [61]. However, the number of missing predictor values was small (<2%), and our post hoc sensitivity analysis showed no major differences in results when a post hoc imputation procedure was performed (S1 and S2 Tables). This suggests that our a priori decision to remove cases with missing predictor values did not bias the results. Finally, because our prognostic model is in the form of a logistic regression equation, this limits its ease of use. To address this limitation, we developed a calculator (based on the recalibrated PICKUP) that is freely available online at http://pickuptool.neura.edu.au/.

Deciding whether a model is useful or not depends both on its performance and its purpose. In the research setting, discrimination is an important consideration. When such a large number of patients recover with minimal or no intervention, treat all approaches to preventing chronic LBP are inevitably going to be inefficient. Some treatments for LBP, if applied to low-risk patients, may even be harmful. Our models can help discriminate between patients who experience poor outcomes and patients who experience good outcomes, with acceptable performance (AUC > 0.6, likelihood ratios not overlapping). In the external validation sample, patients allocated to the high-risk group (i.e., in the highest quartile of predicted risk) were three times more likely to develop chronic LBP than their medium- or low-risk counterparts (in the middle two and lowest quartiles of predicted risk, respectively). Including only patients with a predicted risk above a 30% in a secondary prevention trial would lead to a net reduction of 40 unnecessary episodes of care (for patients with good outcomes) per 100 patients (Fig 4).

In the clinical setting, calibration is important for providing accurate risk estimates to patients. Our primary prognostic model (PICKUP) demonstrated acceptable calibration (<5% difference between predicted risks and observed proportions of chronic LBP) in seven out of ten risk strata. However, we did observe some miscalibration in the higher risk strata—as predicted risk increased, accuracy decreased and the model overestimated risk (Fig 2). This, along with our negative predictive values above 90% (S4 Table), means that people with lower risk estimates are very unlikely to develop chronic pain, but those with higher risk estimates may still recover quickly. That is, the models are better at ruling out future chronic LBP than ruling it in. However, after recalibration the estimates were almost perfectly calibrated (S1–S3 Figs). With further testing and recalibration, these models have potential to be useful in other clinical settings.

Our decision curve analysis suggested that the primary model is likely to be useful for patients whose decision to pursue further intervention is based on a predicted risk between 12% and 35%. The question that remains is whether these thresholds are clinically relevant. For a range of thresholds under 50% to be considered clinically relevant, the assumption is that patients place more value on detecting an imminent problem (true positive rate) than undergoing unnecessary treatment (false positive rate) [62]. We would suggest that most patients with acute LBP would fall into this category: the consequences of undergoing, for example, an unnecessary course of physiotherapy, are outweighed by the prospect of missing a chance at preventing a long-term problem. However, this assumption rests on the nature of the treatment proposed. If the patient and their physician are considering invasive treatments such as spinal surgery, the patient might weigh the false positive rate more heavily, due to the higher risk of adverse events. In this case, a screening tool would need to yield a net benefit across a range of predicted risk cutoffs higher than 50%, and our model would not be considered useful [62]. We therefore speculate that our models are likely to be useful only for informing the choice between a wait-and-see approach and a course of conservative intervention.

Although several models have been developed in LBP, few have been externally validated [21], and none have been designed to predict the onset of chronic LBP. Pain is arguably the most important outcome to predict in LBP; it is clearly the most important issue for patients [63], and it is the slowest to recover [33]. The three available tools that have been tested in external samples of patients with acute LBP appear to predict pain outcomes at 3 and 6 mo with modest accuracy at best. Grotle et al. [31] tested the OMPQ in an acute LBP sample and reported an AUC for predicting pain at 6 mo of 0.62 (95% CI 0.51 to 0.73). Recent evaluations of SBT score in predicting ongoing pain at 6 mo in acute LBP samples reported AUC values of 0.50 [29] and 0.54 [30]. Williams et al. [64] reported an AUC of 0.60 (95% CI 0.56 to 0.64) for predicting recovery from pain (0 or 1/10 pain sustained for 7 d) at 3 mo. PICKUP appears to discriminate medium-term pain outcomes in patients with acute LBP more accurately than other validated models, and may be particularly useful for secondary prevention trials that target pain reduction. Because calibration performance has not been widely reported, we were unable to compare our model to others in these terms. Williams et al. [64] reported acceptable calibration for their model predicting outcomes in the first 2 wk but relatively poor calibration (more than 10% difference between predicted risks and observed proportions) for predicting pain outcomes at 3 mo. As suggested by PROGRESS, a formal comparison of our tool with other validated tools, for example, using a decision curve analysis, is a logical next step.

### Conclusions

Based on its performance in these cohorts, this five-item prognostic model for patients with acute LBP may be a useful tool for estimating risk of chronic LBP. Further validation is required to determine whether screening with this model leads to a net reduction in unnecessary interventions provided to low-risk patients.

## Supporting Information

S1 Data Dataset containing baseline values, predicted risks, and outcomes in the development and external validation cohorts. (XLSX)

S2 Data Data dictionary to accompany S1 Data. (XLSX)

S1 Fig PICKUP recalibrated. (A) PICKUP performance in the development sample. (B) Recalibrated PICKUP performance in the external validation sample. The distribution of predicted risks is shown at the bottom of each plot, by 3-mo outcome. The triangles indicate observed frequencies by decile of predicted risk. Model performance estimates are provided in the top left of each plot.
(TIF)

S2 Fig Model 2a recalibrated. (A) Model 2a performance in the development sample. (B) Recalibrated Model 2a performance in the external validation sample. The distribution of predicted risk is shown at the bottom of each plot, by 3-mo outcome. The triangles indicate observed frequencies by decile of predicted risk. Model performance estimates are provided in the top left of each plot.
(TIF)

S3 Fig Model 2b recalibrated. (A) Model 2b performance in the development sample. (B) Recalibrated Model 2b performance in the external validation sample. The distribution of predicted risks is shown at the bottom of each plot, by 3-mo outcome. The triangles indicate observed frequencies by decile of predicted risk. Model performance estimates are provided in the top left of each plot.
(TIF)

S1 Table Model specification results using two missing data strategies. Compares analysis where cases missing predictor variables were removed to analysis where predictor values were imputed.
(DOCX)

S2 Table Comprehensive model performance results. Reports all performance indices measured in the development and validation samples. Prespecified “acceptable” levels were published in our protocol [32].
(DOCX)

S3 Table PICKUP performance in different clinical settings (development sample). Sensitivity analysis examining model performance in patients seen in physiotherapy, general practice, and chiropractic settings.
(DOCX)

S4 Table Likelihood ratios and posterior probability estimates in external validation sample. Additional measures of predictive performance in the external validation sample.
(DOCX)

S1 TRIPOD Checklist Adherence to TRIPOD reporting criteria for studies of prediction model development and validation. (DOCX)

## Abbreviations

AUC: area under the receiver operating characteristic curve
LBP: low back pain
OMPQ: Orebro Musculoskeletal Pain Questionnaire
PROGRESS: Prognosis Research Strategy
SBT: Start Back Tool
