# Supplementary figures and images for: Estimating the Risk of Chronic Pain: Development and Validation of a Prognostic Model (PICKUP) for Patients with Acute Low Back Pain
Source: PLoS Med. 2016 May 17;13(5):e1002019. doi: 10.1371/journal.pmed.1002019 (PMC4871494; doi:10.1371/journal.pmed.1002019)

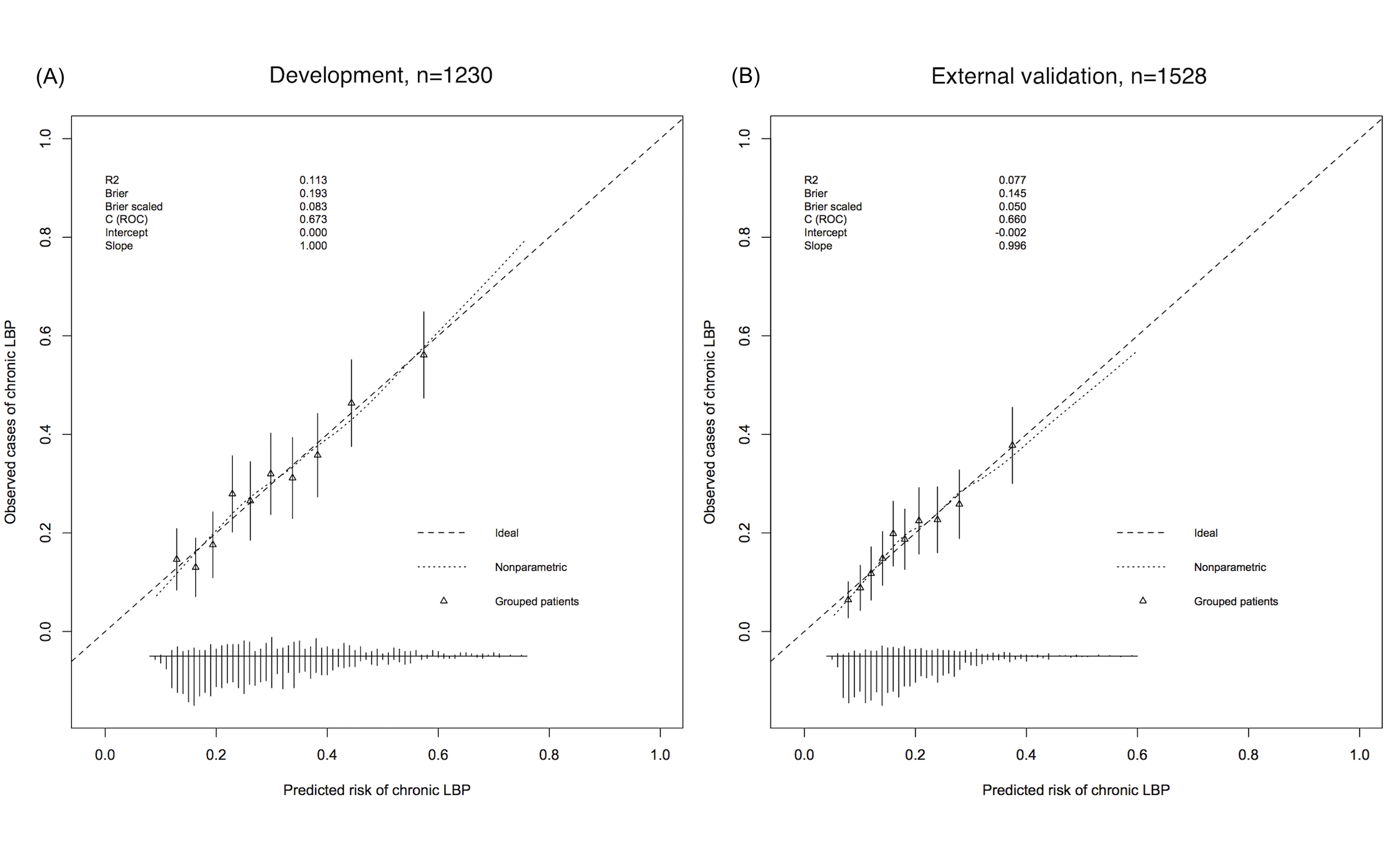

Supplement: S1 Fig — (A) PICKUP performance in the development sample. (B) Recalibrated PICKUP performance in the external validation sample. The distribution of predicted risks is shown at the bottom of each plot, by 3-mo outcome. The triangles indicate observed frequencies by decile of predicted risk. Model performance estimates are provided in the top left of each plot. (TIF) [file pmed.1002019.s003.tif]

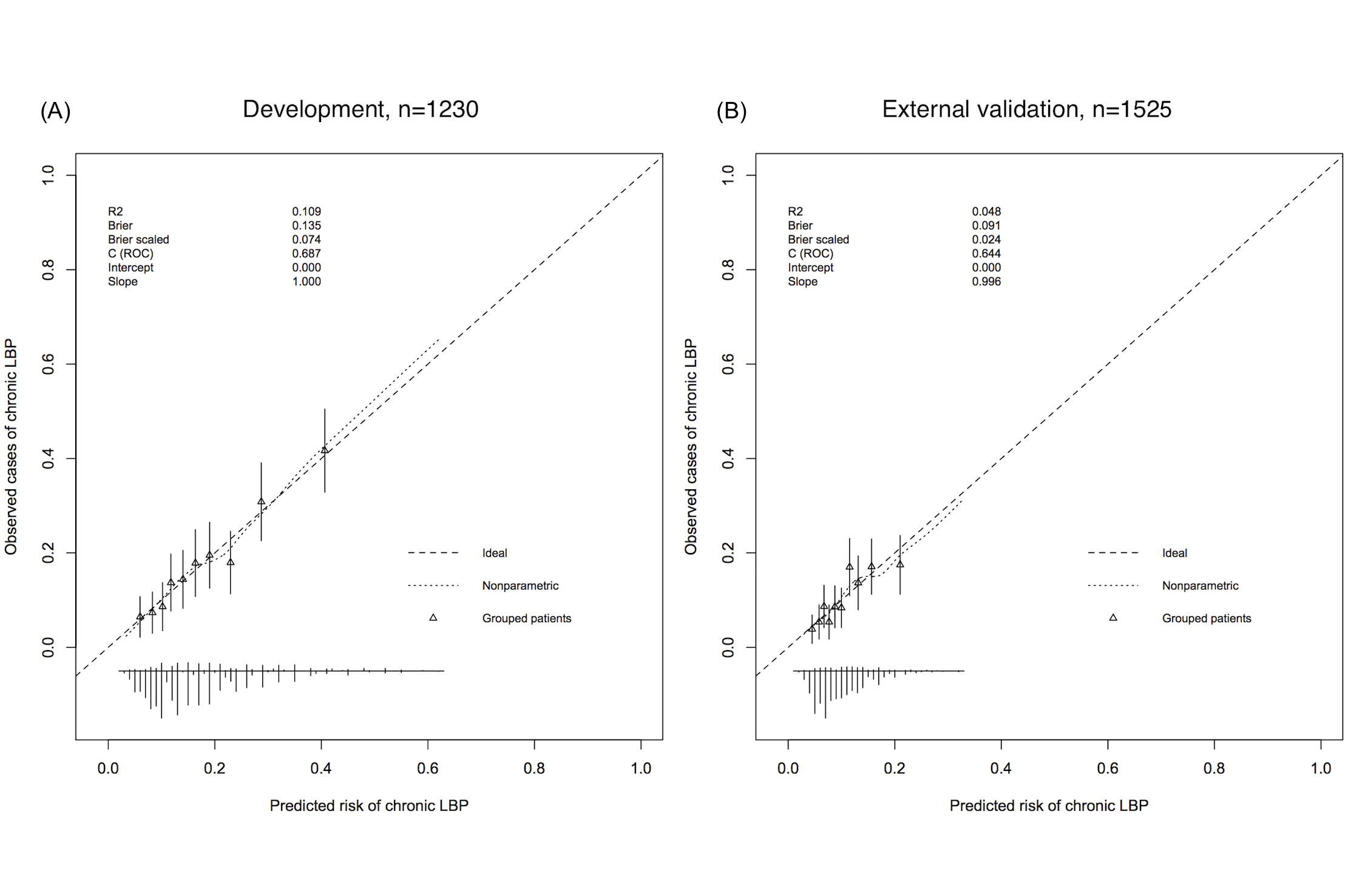

Supplement: S2 Fig — (A) Model 2a performance in the development sample. (B) Recalibrated Model 2a performance in the external validation sample. The distribution of predicted risk is shown at the bottom of each plot, by 3-mo outcome. The triangles indicate observed frequencies by decile of predicted risk. Model performance estimates are provided in the top left of each plot. (TIF) [file pmed.1002019.s004.tif]

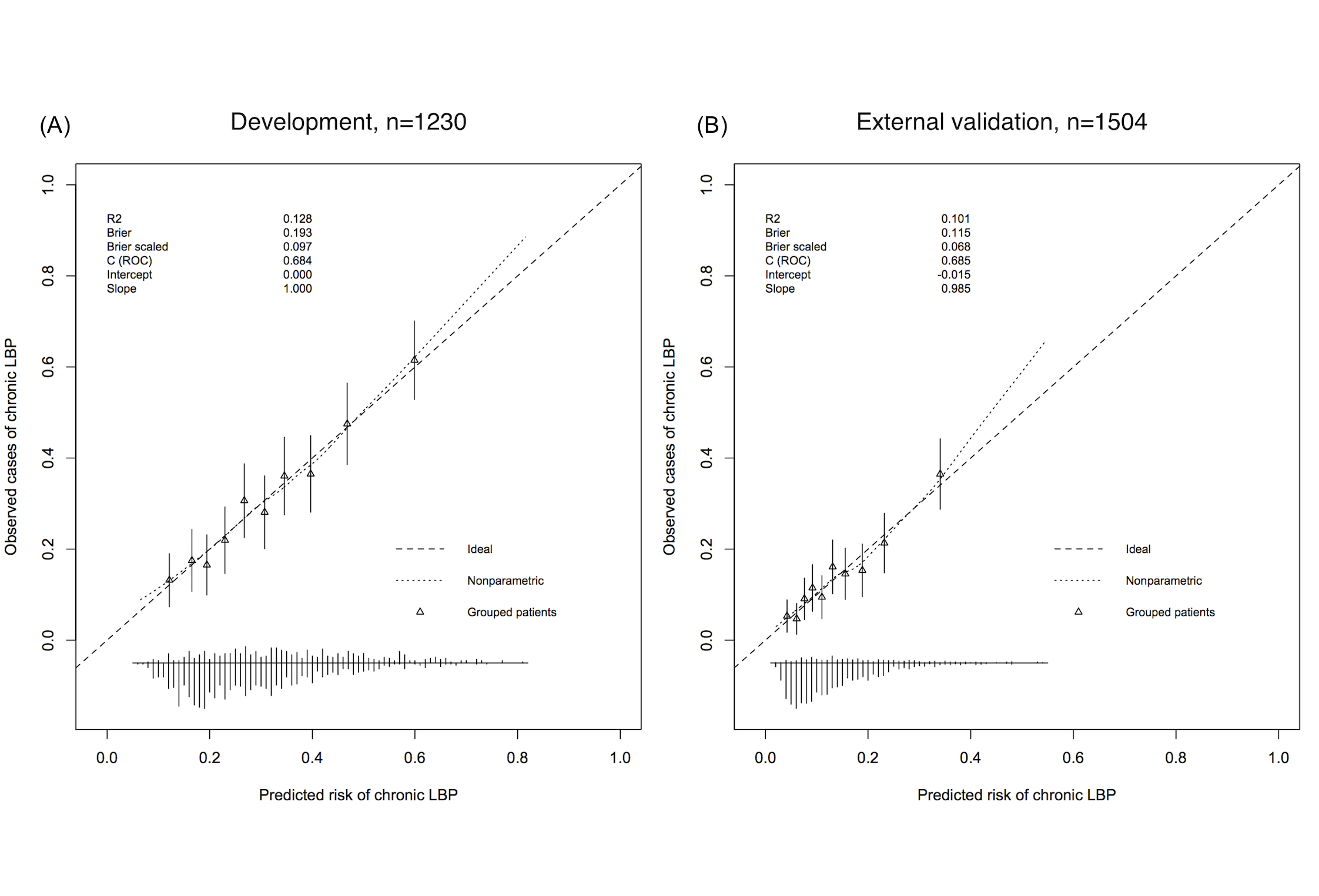

Supplement: S3 Fig — (A) Model 2b performance in the development sample. (B) Recalibrated Model 2b performance in the external validation sample. The distribution of predicted risks is shown at the bottom of each plot, by 3-mo outcome. The triangles indicate observed frequencies by decile of predicted risk. Model performance estimates are provided in the top left of each plot. (TIF) [file pmed.1002019.s005.tif]
