# Supplementary material for: Estimating the Risk of Chronic Pain: Development and Validation of a Prognostic Model (PICKUP) for Patients with Acute Low Back Pain
Source: PLoS Med. 2016 May 17;13(5):e1002019. doi: 10.1371/journal.pmed.1002019 (PMC4871494; doi:10.1371/journal.pmed.1002019)
Supplement: S1 Table — Compares analysis where cases missing predictor variables were removed to analysis where predictor values were imputed. (DOCX) [file pmed.1002019.s006.docx]

|  | PICKUP | | | | Model 2a | | | | Model 2b | | | |
| --- | --- | --- | --- | --- | --- | --- | --- | --- | --- | --- | --- | --- |
| Variable | Complete Case | | Missing Predictor Values Imputed | | Complete Case | | Missing Predictor Values Imputed | | Complete Case | | Missing Predictor Values Imputed | |
|  | Regression coefficient | Odds ratio (95% CI) | Regression coefficient | Odds ratio (95% CI) | Regression coefficient | Odds ratio (95% CI) | Regression coefficient | Odds ratio (95% CI) | Regression coefficient | Odds ratio (95% CI) | Regression coefficient | Odds ratio (95% CI) |
| Compensation status (y/n) | 0.50 | 1.65 (1.20 to 2.25) | 0.59 | 1.81 (1.31 to 2.5) | 0.42 | 1.52 (1.06 to 2.18) | 0.50 | 1.66 (1.14 to 2.41) | 0.43 | 1.53 (1.12 to 2.09) | 0.51 | 1.67 (1.21 to 2.31) |
| Leg pain (y/n) | 0.44 | 1.56 (1.17 to 2.08) | 0.35 | 1.42 (1.06 to 1.91) | 0.53 | 1.71 (1.23 to 2.38) | 0.44 | 1.55 (1.10 to 2.17) | 0.46 | 1.58 (1.18 to 2.10) | 0.38 | 1.46 (1.08 to 1.96) |
| Pain intensity (1-6 scale) | 0.21 | 1.23 (1.06 to 1.44) | 0.23 | 1.26 (1.07 to 1.48) | 0.28 | 1.32 (1.09 to 1.60) | 0.29 | 1.33 (1.09 to 1.63) | 0.25 | 1.29 (1.10 to 1.50) | 0.27 | 1.32 (1.12 to 1.56) |
| Depression (0-10 scale) | 0.06 | 1.06 (1.02 to 1.11) | 0.06 | 1.06 (1.01 to 1.11) | NS | NS | NS | NS | 0.07 | 1.07 (1.03 to 1.12) | 0.06 | 1.07 (1.02 to 1.11) |
| Self perceived risk (0-10 scale) | 0.13 | 1.14 (1.09 to 1.20) | 0.12 | 1.12 (1.08 to 1.19) | 0.14 | 1.15 (1.09 to 1.22) | 0.13 | 1.14 (1.08 to 1.21) | 0.11 | 1.12 (1.07 to 1.17) | 0.10 | 1.11 (1.06 to 1.17) |
| Medication use (y/n) | NS | NS | NS | NS | 0.40 | 1.49 (1.08 to 2.05) | 0.38 | 1.46 (1.05 to 2.01 | NS | NS | NS | NS |
| General health (1-5 scale) | NS | NS | NS | NS | NS | NS | NS | NS | 0.25 | 1.28 (1.10 to 1.48) | 0.23 | 1.26 (1.08 to 1.96) |
| Constant | -2.82 |  | -3.90 |  | -3.92 |  | -4.95 |  | -3.49 |  | -4.44 |  |

**S1 Table. Model specification results using 2 missing data strategies.**
