# Supplementary material for: Estimating the Risk of Chronic Pain: Development and Validation of a Prognostic Model (PICKUP) for Patients with Acute Low Back Pain
Source: PLoS Med. 2016 May 17;13(5):e1002019. doi: 10.1371/journal.pmed.1002019 (PMC4871494; doi:10.1371/journal.pmed.1002019)
Supplement: S2 Table — Reports all performance indices measured in the development and validation samples. Prespecified “acceptable” levels were published in our protocol [32]. (DOCX) [file pmed.1002019.s007.docx]

**S2 Table. Comprehensive model performance results.**

|  | |  | PICKUP | | | Model 2a | | | Model 2b | | |
| --- | --- | --- | --- | --- | --- | --- | --- | --- | --- | --- | --- |
| Aspect | Measure | Acceptable performance^a^ | Development^b^  (N=1230) | External validation | | Development^b^  (N=1230) | External validation | | Development^b^  (N=1230) | External validation | |
|  |  |  |  | Complete case (N=1528) | Missing predictor values imputed (N=1541) |  | Complete case (N=1525) | Missing predictor values imputed (N=1544) |  | Complete case (N=1504) | Missing predictor values imputed (N=1519) |
| Overall Performance | *R*^2^ (Nagelkerke) | Not pre-specified | 10.9 | 7.7 | 7.9 | 9.7 | 4.8 | 5.0 | 11.7 | 10.1 | 10.1 |
|  | Brier score | <0.25 | 0.19 | 0.15 | 0.15 | 0.14 | 0.09 | 0.10 | 0.19 | 0.13 | 0.13 |
| Discrimination | AUC | >0.6 | 0.67 (0.64 to 0.70) | 0.66 (0.63 to 0.69) | 0.66 (0.63 to 0.70) | 0.68 (0.65 to 0.73) | 0.64 (0.60 to 0.68) | 0.65 (0.60 to 0.69) | 0.67 (0.65 to 0.72) | 0.69 (0.64 to 0.72) | 0.69 (0.65 to 0.72) |
|  | Discrimination slope | Not pre-specified | 0.08 | 0.07 | 0.03 | 0.07 | 0.04 | 0.02 | 0.1 | 0.1 | 0.04 |
| Calibration | Calibration intercept | Close to 0 | -0.02 | -0.55 | -0.52 | -0.05 | -0.81 | -0.77 | -0.02 | -0.86 | -0.86 |
|  | Calibration slope | Close to 1 | 0.98 | 0.89 | 0.90 | 0.95 | 0.74 | 0.75 | 0.96 | 0.99 | 0.98 |
|  | Hosmer-Lemeshow | p>0.05 | χ^2^ = 4.64  p = 0.79 | χ^2^ =6.54  p =0.59 | χ^2^ =2.62  p =0.96 | χ^2^ = 3.30  p = 0.91 | χ^2^ =11.42  p =0.18 | χ^2^ =13.03  p =0.11 | χ^2^ = 3.58  p = 0.89 | χ^2^ =6.32  p =0.61 | χ^2^ =7.95  p =0.44 |

^a^ pre-specified in our published protocol

^b^ bootstrapped (optimism adjusted) estimate
