# Supplementary material for: Estimating the Risk of Chronic Pain: Development and Validation of a Prognostic Model (PICKUP) for Patients with Acute Low Back Pain
Source: PLoS Med. 2016 May 17;13(5):e1002019. doi: 10.1371/journal.pmed.1002019 (PMC4871494; doi:10.1371/journal.pmed.1002019)
Supplement: S3 Table — Sensitivity analysis examining model performance in patients seen in physiotherapy, general practice, and chiropractic settings. (DOCX) [file pmed.1002019.s008.docx]

**S3 Table. PICKUP performance in different clinical settings (development sample).**

| Setting | Physical therapy (N=951) | General practice (N=247) | Chiropractic (N=50) |
| --- | --- | --- | --- |
| R^2^ | 11.4 | 15 | 0.02 |
| AUC (95%CI) | 0.67 (0.64 to 0.71) | 0.70 (0.63 to 0.76) | 0.57 (0.26 to 0.60) |
| Calibration intercept | -0.01 | 0.29 | -1.50 |
| Calibration slope | 1.04 | 1.10 | -0.41 |
