# Supplementary material for: Estimating the Risk of Chronic Pain: Development and Validation of a Prognostic Model (PICKUP) for Patients with Acute Low Back Pain
Source: PLoS Med. 2016 May 17;13(5):e1002019. doi: 10.1371/journal.pmed.1002019 (PMC4871494; doi:10.1371/journal.pmed.1002019)
Supplement: S4 Table — Additional measures of predictive performance in the external validation sample. (DOCX) [file pmed.1002019.s009.docx]

**S4 Table. Likelihood ratios and posterior probability estimates in external validation sample.**

| Model | PICKUP | | Model 2a | | Model 2b | |
| --- | --- | --- | --- | --- | --- | --- |
| Chronic LBP definition | >=3/10 Pain | | >=5/10 Pain | | >=7/24 RDQ | |
| Incidence | 18.7% | | 9.8% | | 14.4% | |
| Risk group | High vs Medium/Low | High/Medium vs Low | High vs Medium/Low | High/Medium vs Low | High vs Medium/Low | High/Medium vs Low |
| LR+ | 2.99 (2.81 to 3.18) | 1.99 (1.93 to 2.05) | 2.44 (2.32 to 2.57) | 1.80 (1.75 to 1.84) | 3.52 (3.28 to 3.75) | 2.39 (2.39 to 2.40) |
| LR- | 0.52 (0.51 to 0.53) | 0.35 (0.34 to 0.36) | 0.53 (0.52 to 0.54) | 0.36 (0.35 to 0.37) | 0.45 (0.44 to 0.46) | 0.34 (0.34 to 0.35) |
| Posterior probabilities |  |  |  |  |  |  |
| PPV | 0.34 (0.28 to 0.40) | 0.24 (0.21 to 0.27) | 0.18 (0.14 to 0.23) | 0.13 (0.11 to 0.15) | 0.25 (0.21 to 0.30) | 0.18 (0.15 to 0.20) |
| NPV | 0.84 (0.82 to 0.86) | 0.90 (0.88 to 0.93) | 0.92 (0.89 to 0.93) | 0.94 (0.92 to 0.96) | 0.90 (0.88 to 0.92) | 0.94 (0.91 to 0.95) |

LR+ = positive likelihood ratio, LR- = negative likelihood ratio, PPV = positive predictive value, NPV = negative predictive value
